# BeeGees: A High‐Throughput Protein‐Coding DNA Barcode Recovery Pipeline Tailored for Genome Skims of Museum Specimens

**DOI:** 10.1111/1755-0998.70170

**Published:** 2026-06-19

**Authors:** Daniel A. J. Parsons, Rutger A. Vos, Benjamin W. Price

**Affiliations:** ^1^ Natural History Museum London UK; ^2^ Naturalis Biodiversity Center Leiden the Netherlands

**Keywords:** barcoding, biodiversity genomics, genome skims, historical DNA, museomics

## Abstract

Natural history collections are unparalleled archives of global biodiversity, yet most specimens remain molecularly uncharacterised due to the technical challenges of historical DNA (hDNA), including degradation, low endogenous content and contamination. Genome skimming offers a scalable alternative to PCR‐based barcoding, but existing bioinformatic workflows are not optimised for the heterogeneous, metagenomic nature of museum‐derived data. Here we present BeeGees (Barcode Extraction and Evaluation from Genome Skims), a high‐performance computing (HPC) integrated, Snakemake‐based workflow designed for protein‐guided recovery and validation of mitochondrial and plastid barcode genes from degraded short‐read genome sequences. BeeGees integrates dual read pre‐processing, systematic per‐sample parameter sweeps, sequential consensus cleaning to remove contaminant sequences and rigorous structural and taxonomic validation against curated reference databases. We benchmarked BeeGees on 1518 museum specimen‐derived genome skims spanning eight phyla. The workflow completed in approximately 120 h (< 5 min per sample) on HPC infrastructure. When excluding sequencing failures (< 1 M reads), validated COI barcodes were recovered for 73.2% of specimens (1050/1435). Barcode recovery success was influenced by endogenous content, preservation quality and parameter choice rather than raw read count alone, highlighting the importance of systematic parameter optimisation. Sequential consensus cleaning eliminated ambiguous bases and reduced chimeric artefacts, proving essential for robust museomic analyses. BeeGees provides a reproducible, scalable framework for high‐throughput barcode recovery and biodiversity genomics and reference gap‐filling initiatives from natural history collections. The BeeGees pipeline is available at: https://github.com/bge‐barcoding/BeeGees/.

## 1 Introduction

Natural history collections serve as irreplaceable archives of Earth's biodiversity, preserving specimens that define species, document their distributions, their evolutionary relationships and ecological changes over time. These collections are increasingly recognised as critical resources for addressing contemporary challenges in biodiversity research (e.g., Marsh et al. 2025). However, sequencing historical DNA (hDNA) from museum specimens (i.e., museomics), which can be decades or centuries old, presents significant technical challenges due to DNA degradation, low DNA concentration, contamination due to sample handling and shared storage and presence of inhibitors within the tissue or the preservatives used (Kapp et al. 2021; Mullin et al. 2023).

Conventional PCR‐based Sanger sequencing of standard barcoding loci, such as mitochondrial cytochrome c oxidase subunit I (COI) in animals and plastid Ribulose‐1,5‐bisphosphate carboxylase/oxygenase (rbcL/RuBisCo) and Maturase K (MatK) in plants, have long underpinned DNA‐based identification (Hebert et al. 2003; CBOL Plant Working Group et al. 2009) and account for the majority of sequences in public repositories. However, when applied to historical specimens these approaches require taxon‐specific primers for short amplicons and repeated amplification attempts, often yielding low success with older material (Hebert et al. 2013; Prosser et al. 2016; D'Ercole et al. 2021). In contrast, low‐coverage whole genome sequencing (WGS) or ‘genome skimming’ (typically 0.1–5× genome coverage), offers a scalable, cost‐effective alternative for multiplexed recovery of barcoding genes, high‐copy number regions and organellular genomes from historical specimens, even in highly degraded, low‐concentration samples (Trevisan et al. 2019; Bleidorn et al. 2026; Quattrini et al. 2024).

Several pipelines have been developed to enable gene and organelle recovery from genome skims, including skim2mito (White et al. 2025), MITOCOMP (SamLMG 2025, https://github.com/SamLMG/MitoComp), PhyloHerb (Cai et al. 2022), ORTHOSKIM (Pouchon et al. 2022) and GeneMiner2 (Yu et al. 2026), although few are specifically designed for museomics and therefore are accompanied by limitations in the context of low‐coverage museum‐derived genomic data. These workflows typically reconstruct organellar genomes using nucleotide‐based assembly or mapping nucleotide reads to closely related nucleotide references. While effective in most contexts, these approaches remain constrained when applied to museomics, where short read lengths, degraded templates, suboptimal database representation and taxonomic divergence often result in incomplete or failed assemblies, and thus reduced barcode/organelle recovery efficiency.

Contrastingly, tools such as MitoGeneExtractor (MGE) which employ protein‐guided alignments to extract target protein‐coding genes (PCGs) mitigate some of these limitations by leveraging the redundancy of the genetic code and aligning reads at the amino acid (aa) level, allowing use of more distantly related references and improving tolerance to degradation and divergence (Brasseur et al. 2023). This is a particular advantage when attempting to address taxonomic gaps at scale. Although MGE is therefore suited for the task at hand, it lacks integrated per‐sample reference optimisation, read quality control prior to alignment and is not yet optimised for the metagenomic nature of hDNA‐derived genome‐skim sequence data, where issues such as uneven coverage, short read lengths and exogenous contamination confound barcode recovery.

Recognising the increasing necessity of biodiversity genomics to aid conservation efforts, the Biodiversity Genomics Europe (BGE) consortium has identified the development of standardised, accessible workflows as a critical priority for unlocking the molecular potential of European museum collections (Koureas et al. 2026). With an estimated 1.5 billion specimens housed across European institutions (European Commission report 2021), the scale of potential molecular data recovery far exceeds what can be processed using current bioinformatic approaches. Meeting this challenge requires robust, reproducible pipelines that can accommodate variable‐quality museomic data while delivering accurate barcode sequences at scale, in line with established standards.

**FIGURE 1:**
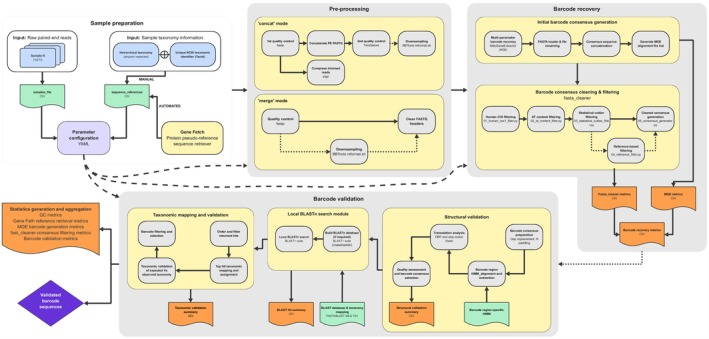
Overview of BeeGees workflow, highlighting the primary processes within sample preparation, dual PE raw read pre‐processing modes, initial multi‐parameter barcode recovery and ‘cleaning’ of generated barcode consensus sequences using the fasta_cleaner module, and then final barcode validation.

To this end, Barcode Extraction and Evaluation from Genome Skims (BeeGees), a High‐Performance Computing (HPC) cluster‐integrated, Snakemake‐based workflow (Köster and Rahmann 2012), was designed specifically for extracting mitochondrial and plastid protein‐coding genes from paired‐end (PE) or single‐read (SE) Illumina genome skim sequence data derived from museum specimens (https://github.com/bge‐barcoding/BeeGees). Barcoding genes are directly recovered from fragmented reads via protein reference‐sequence alignments, utilising a dual pre‐processing strategy and multi‐parameter barcode extraction to maximise recovery success. The pipeline addresses the unique challenges of hDNA processing, incorporating contamination detection, automated barcode gene extraction across multiple parameter combinations, structural and taxonomic validation, selection of optimal consensus sequences and comprehensive quality reporting (Figure 1).

To demonstrate its utility, the BeeGees pipeline was used to recover (658 bp) COI barcode sequences from 1518 genome‐skim datasets derived from museum specimens sequenced within the BGE project. This large‐scale test demonstrates BeeGees' robustness, sensitivity and scalability, establishing it as a valuable tool for high‐throughput protein‐coding barcode recovery from natural history collections.

## 2 Methods

### 2.1 Workflow Overview

Broadly, BeeGees includes raw sequence data pre‐processing, barcoding gene recovery, barcode consensus cleaning and structural and taxonomic barcode validation. The pipeline supports dynamic resource allocation, memory scaling across workflow modules, automatic error handling, workflow resumption and dependency tracking using Snakemake (version 9.9.0), enabling efficient and flexible utilisation of HPC (High Performance Computing) clusters. Configuration is controlled via editable parameters and specifying metadata files, allowing easy adjustment of quality thresholds, taxonomic parameters and resource allocation. Software dependencies are managed through Conda (version 24.11.3), with a pre‐configured environment provided.

### 2.2 Input Requirements

To run the BeeGees workflow, only two inputs are required: (1) a YAML configuration file outlining run parameters and filepaths; (2) a sample metadata file that includes filepaths to the PE Illumina format reads in FASTQ format (compressed or uncompressed), unique sample identifiers and NCBI taxonomic IDs (taxid) or sample taxonomic hierarchy. Optionally, a reference amino acid sequence of the target gene for each sample can be provided manually by the user or retrieved within the pipeline using the integrated ‘Gene Fetch’ tool (Parsons and Price 2025).

### 2.3 Dual Pre‐Processing of Raw Genome Skim Data

To accommodate degraded and variable‐length DNA typical of historical specimens (Mullin et al. 2023), BeeGees implements parallel modes for preprocessing and quality control of each sample's raw sequence data: Concatenation mode (‘concat’) and Merge mode (‘merge’).

Both modes utilise fastp for default quality and length filtering, Illumina TruSeq adapter removal, poly‐G tail trimming and deduplication (Chen et al. 2018). In ‘merge’ mode, fastp also performs PE read merging and error correction in a single step, as merging overlapping regions of PE reads can yield longer read lengths and increase confidence through error correction (Lien et al. 2023). However, this approach discards reads that cannot be merged (i.e., unmerged reads), thereby reducing the available sequence data for downstream analysis. Retaining unmerged reads may enhance results when working with limited but higher‐quality sequence data (Lien et al. 2023), although such reads may also represent longer fragments of modern contaminant DNA and so unmerged reads are omitted here. In ‘concat’ mode, forward and reverse reads are instead combined into a single file for additional quality filtering with Trim Galore (utilising Cutadapt to remove terminal degradation artefacts and extremely short sequences (< 20 base pairs (bp))), making it suitable for shorter, lower‐quality reads where merging may not be feasible (Martin 2011; Krueger 2024). After quality control, concatenated PE and merged read data can be optionally downsampled using BBTools' reformat.sh (Bushnell 2014), thereby standardising the number of reads used for barcode recovery across samples, if desired by the user.

### 2.4 Protein‐Guided Barcode Gene Recovery

Subsequent barcode gene recovery is attempted in parallel using trimmed and filtered reads from both preprocessing modes. This approach avoids assumptions about input read quality and structure, allowing selection of the optimal consensus sequence for each sample.

Employing MitoGeneExtractor (MGE), the pipeline extracts target PCGs using an amino acid‐guided alignment, via Exonerate (Slater and Birney 2005), which aligns translated nucleotide reads to sample‐specific protein pseudo‐reference sequences. This protein‐guided approach is particularly effective for fragmented DNA, where reads are ‘baited’ onto the reference at the protein level and then translated back to nucleotide sequences to form the barcode consensus sequence (Brasseur et al. 2023).

Configuration of the BeeGees pipeline allows the application of multiple MGE parameter combinations to each sample to optimise recovery and compensate for variable read numbers and length distributions across samples. The Exonerate relative score threshold (‘r’) and minimum score threshold (‘s’) work together as complementary quality filters, where ‘r’ normalises alignment scores by length (score divided by alignment length, with reasonable values between 0.7 and 2.0) and ‘s’ sets an absolute minimum exonerate score cutoff (Brasseur et al. 2023). Higher values for both parameters increase stringency, retaining fewer reads with higher‐quality alignments. In comparison, lower values allow more permissive matching to enable alignment of shorter reads, but may capture ‘weaker’, spurious alignments. Since sequences must pass both thresholds simultaneously, this dual filtering approach enables fine‐tuned control over alignment across samples of varying quality.

Additional parameters include specifying genetic code (‘C’) for appropriate translation during alignment and the consensus threshold (‘t’), which determines the minimum base frequency required for a nucleotide to be called in the final consensus sequence (e.g., 0.5 = 50%).

### 2.5 Consensus Cleaning

After initial consensus generation, the fasta_cleaner module applies sequential filtering to remove low‐quality sequences, statistical outliers and potential contaminants from each MGE‐generated barcode consensus sequence, producing ‘cleaned’ consensus sequences. Because hDNA‐derived reads are typically short (Mullin et al. 2023), it is necessary to lower the ‘s’ and ‘r’ values within MGE. However, this can increase the risk of aligning poorly matching longer sequences when contaminants are present.

Human contamination is first identified by per‐base similarity comparison against a human COI reference sequence (MZ920758.1), with reads exceeding a configurable threshold (e.g., ≥ 95% similarity) removed (‘01_human_coi_filter.py’). Second, non‐target read filtering is employed using AT (%) ratio comparisons by generating an internal consensus sequence and calculating AT nucleotide composition for both the consensus and aligned reads (‘02_at_content_filter.py’). As AT nucleotide composition varies across taxa and genome regions, reads deviating substantially from the consensus AT content are likely to represent non‐target contamination rather than genuine target‐derived sequences. Sequences are filtered using three configurable modes: ‘absolute’ removes reads differing from consensus AT content over a set threshold (e.g., > 10%) in either direction, whilst ‘higher’ and ‘lower’ modes filter in one direction only, enabling targeted removal of sequences with aberrant nucleotide composition indicative of contamination (Kumar et al. 2013).

Aligned reads identified as statistical outliers are then removed using a dual‐scoring approach that evaluates each sequence against an internally generated consensus (‘03_statistical_outlier_filter.py’). Two complementary metrics are calculated, an unweighted score representing the simple proportion of mismatches at comparable positions, and a weighted score in which mismatches are penalised according to the conservation of each site, such that deviations at highly conserved positions contribute disproportionately. Sequences exceeding a configurable percentile threshold (e.g., 90th) in either metric are flagged as outliers and removed, filtering potential sequencing errors, paralogs or residual contaminants.

Next, an optional BWA‐based reference‐filtering step enables selective removal or retention of sequences based on alignment to user‐specified nucleotide references (‘04_reference_filter.py’). The ‘remove_similar’ mode filters sequences mapping to provided references (e.g., closely related species, expected contaminants, NUMTs, or host DNA), whilst ‘keep_similar’ retains only sequences mapping to references, enabling targeted retention of specific genetic markers or taxonomic groups. Finally, cleaned consensus sequences are generated from the sequentially filtered alignments (‘05_consenus_generator.py’) using position‐wise majority‐rule with a configurable consensus threshold (e.g., 50%).

Comprehensive quality metrics are then calculated for each cleaned consensus sequence, appended with ‘fcleaner’, at each cleaning step (‘06_aggregate_filter_metrics.py’ and ‘barcode_consensus_count.py’).

### 2.6 Barcode Validation and Selection

Consensus sequences initially generated via MGE‐based barcode recovery and cleaned with the fasta_cleaner (fcleaner) module are subsequently validated and selected using internal structural and taxonomic validation modules following criteria outlined in Barcode Validator (Vos et al. under review).

Structural validation first calculates full‐sequence quality metrics for each consensus, including sequence length, terminal (‘‐’) and internal gap (‘~’) counts, ambiguous base (N) count and longest contiguous segment without gaps or Ns (‘structural_validation.py’). Sequences are then aligned against hidden Markov models (HMMs) representing the barcoding region of the target gene using nhmmer (Wheeler and Eddy 2013). At release, HMMs representing the barcoding regions of COI‐5P and rbcL are supported. HMM‐alignment quality (e‐value, score and bias) and mapping coordinates in HMM space are extracted for each consensus sequence. Alignments meeting an e‐value threshold of ≤ 1 × 10^−3^ are retained, with aligned segments positioned within HMM coordinate space and unaligned positions converted to Ns to maintain positional information whilst highlighting regions of uncertainty. Leading and trailing Ns are trimmed whilst preserving internal Ns that reflect true (i.e., original) ambiguity or structural features. Barcode region‐specific metrics are then calculated, including total length, final N count and informative base count (total barcode length − *N* count). Translation analysis is subsequently performed across all three reading frames using a configurable genetic code table, with complete codons translated to protein sequences. Lastly, sequences are ranked on a six‐tier scale based on BOLD's BIN (Barcode Index Number) criteria (Ratnasingham and Hebert 2013), with barcodes filtered to retain those with no original N's (indicative of chimeras), no stop codons, valid reading frames, > 300 bp informative base count and < 30% N count in the final barcode. Barcodes passing these criteria are taken forward for taxonomic validation and selection of the best consensus sequence for each sample. This systematic approach ensures only high‐quality barcode sequences proceed to taxonomic validation.

Taxonomic validation uses local BLASTn searches against a specified reference database, followed by hierarchical taxonomy matching to validate the expected against the observed taxonomy (‘tv_local_blast.py’ and ‘tv_blast2taxonomy.py’). Reference databases can be input as existing BLAST databases or FASTA files (Camacho 2024). BLASTn is executed with configurable parameters (default: e‐value ≤ 1 × 10^−5^, maximum 500 targets) against taxonomically annotated databases, such as BOLDistilled for COI (Prosser et al. 2025) or a curated database for rbcL (Dubois et al. 2022).

Hierarchical taxonomy matching is performed between the expected (input) taxonomic lineage and the BLAST hits (observed) for each barcode, at the family, genus and species ranks. Hits below configurable identity and alignment length thresholds are excluded, while remaining hits that match the expected lineage are retained. Among these sequences, the optimal representative for each sample is selected based on taxonomic specificity (species > genus > family), followed by alignment quality (percent identity, mismatches, gaps, e‐value, alignment length). If necessary, final tie‐breaking prioritises sequences recovered using higher MGE parameters (‘s’ and ‘r’ values) and cleaned (‘fcleaner’) barcodes, as initial testing found that lower MGE parameters, particularly ‘s’, could sometimes result in chimeric alignments and inclusion of contaminant reads.

The validation process generates comprehensive outputs that include taxonomy‐matching outcomes (YES/NO), matched rank, hit‐quality statistics and parsed taxonomies for the top 10 quality‐ranked hits, providing taxonomic context for manual review (‘barcoding_outcome.py’). A FASTA file containing the successfully validated barcodes for each sample is subsequently output for downstream analyses, and structural and taxonomic validation statistics, as well as metrics from the preceding steps of the BeeGee pipeline (‘compile_barcding_statistics.py’), are then merged into a single, consolidated spreadsheet (‘val_csv_merger.py’).

### 2.7 Benchmarking Dataset, Sampling, Sequencing and Parameter Configuration

A total of 1518 genome skims generated from museum specimens, encompassing 8 phyla, 18 classes, 54 orders and 212 families, were selected for analysis based on a single batch of sequenced samples within the BGE project (Tables S1 and S2). The sampled specimens were ‘pinned’ and ethanol‐preserved, depending on the taxa, with collection dates ranging from 1896 to 2024.

The samples were prepared following Price (2025), with the lab procedure outlined in Marsh et al. (2025): DNA extractions following Hall et al. (2023), library preparations based on the Santa Cruz Reaction (SCR) protocol by Kapp et al. (2021) and modifications by Nguyen et al. (2023). The reaction volumes were halved from those reported in the original publications to reduce reagent costs, with additional minor modifications documented in the SOP (Price et al. 2025). Following equimolar pooling of four plates into each pool, the samples were sequenced (2 × 150 bp) on lanes of an Illumina NovaSeq 25B at the SNP&SEQ Technology Platform in Uppsala, Sweden.

BeeGees was run using Gene Fetch with ‘taxid’ input, retrieving COI protein sequences (> 500 aa in length) for use as sample‐specific pseudo‐references. Six MGE parameter combinations were provided (*r* = 1, 1.3, 1.5; s = 50, 100). Downsampling was disabled so that all reads were processed. The fasta_cleaner module applied default thresholds (≥ 50% consensus agreement, ≥ 95% human COI similarity removal, ≤ 10% absolute AT‐content difference, statistical outlier removal), excluding optional reference‐based filtering. Together with the two (‘concat’ and ‘merge’) pre‐processing modes, 6 MGE parameter combinations and fasta_cleaner module, up to 24 consensus sequences were generated per sample, which underwent structural validation (COI‐5P HMM, genetic code 5) and taxonomic validation against BOLDistilled (July 2025; Prosser et al. 2025), requiring ≥ 80% identity over ≥ 100 bp at a minimum of family‐level identification. All analyses were performed on the UKCropDiversity high‐performance computing facility (Percival‐Alwyn et al. 2025). Jobs were submitted via a SLURM profile with a maximum of 20 concurrent jobs, where per‐job resource allocations ranged from 4 to 32 threads and 4–50 GB RAM depending on the rule, with the most demanding steps (MitoGeneExtractor, structural and taxonomic validation) allocated 8–32 threads and 32–50 GB RAM.

### 2.8 Statistical Analysis of Barcode Recovery Predictors

To identify factors associated with barcode recovery success, a binomial logistic regression model was fitted in R using the glm() function (v4.4.1; R Core Team 2024). Barcode recovery success was treated as the binary response variable, where a barcode was either validated and successfully recovered or it was not. Raw read count was log10‐transformed prior to modelling to account for the multiplicative relationship between raw read count and recovery probability, and specimen collection year was included as a continuous numeric predictor. Taxonomic Order was initially considered as a categorical predictor but was excluded from the final model due to complete separation, where, due to the large number of Orders represented by very few specimens, many levels resulted in no outcome variance and produced numerically unstable coefficient estimates. Model coefficients are reported as odds ratios (OR) with 95% Wald confidence intervals. The analysis was restricted to only include those samples with valid collection date metadata (*n* = 1037).

## 3 Results

The BeeGees workflow executed 27,355 Snakemake jobs to process all genome skims, including 18,216 MGE jobs across all parameter combinations. The workflow took approximately 120 h of wall time to run to completion (< 5 min per sample). Based on Snakemake log‐derived runtimes, this comprised ~20 h read pre‐processing, 9 h for pseudo‐reference retrieval using Gene Fetch, 83 h for barcode recovery, 8 h for consensus filtering and cleaning and 4 h for barcode validation. Excluding sequencing failures, validated barcodes were recovered for 1050 of the 1435 (73.2%) specimens.

### 3.1 Raw Read Counts and Taxonomic Diversity

**FIGURE 2:**
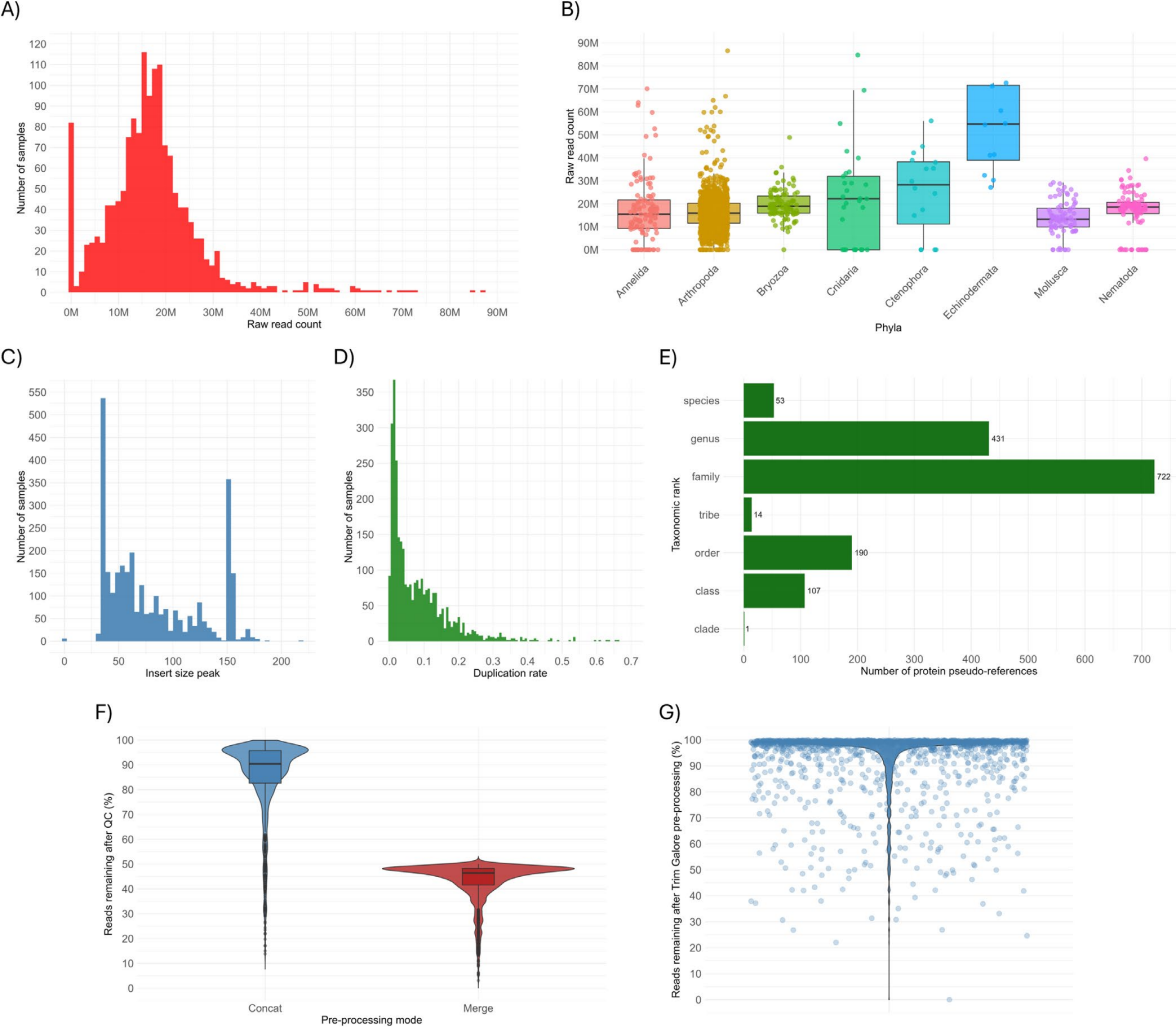
Pre‐processing metrics for 1518 historical DNA genome skims run through the BeeGees pipeline. (A) Distribution of raw read count, (B) Raw read count grouped by taxonomic phylum, (C) Distribution of insert size peak, (D) Distribution of duplication rate, (E) Number of protein pseudo‐references retrieved by Gene Fetch for each taxonomic rank, (F) Percentage of reads remaining for each pre‐processing mode after fastp filtering, and (G) Percentage reads remaining for PE ‘concat’ mode reads after Trim Galore filtering.

Raw read counts varied substantially across the 1518 genome skims, ranging from 2 reads and ~329 million (M) reads, with an average of 17.4 M reads across all skims (Figure 2A,B). This variability extended across the 8 sampled phyla, where arthropoda, the most extensively sampled (*n* = 1075), had an average of 16.6 M raw reads (Figure 2B). Echinodermata (*n* = 12) yielded the highest average read count (64.1 M reads) with the greatest intra‐phylum variation, whilst Mollusca (*n* = 79) produced the lowest (13.9 M reads) (Figure 2B). Of the 1518 samples, 83 exhibited low read counts (< 1 M), were thus classified as sequencing failures and were not expected to generate reliable barcode sequences.

Insert size was characteristically small for historical specimen‐derived data, with an average peak insert size of 82 bp across all skims (Figure 2C). Sequencing duplication rates were also low (average: 0.082%), indicating efficient library preparation and suitable library complexity across samples (Figure 2D).

### 3.2 Protein Pseudo‐Reference Retrieval Using Gene Fetch

Protein reference sequences were retrieved from GenBank for all genome skims using the integrated Gene Fetch module. Each sample‐specific pseudo‐reference corresponded to a cytochrome oxidase I (COI) protein sequence exceeding 500 amino acids (aa) in length (Table S3).

Retrieved protein sequences ranged from 502 to 1067 aa (average: 519.9 aa), which is indicative of consistent retrieval of complete or near‐complete COI protein references across taxa. This range reflects the taxon‐specific variation in COI sequence length as well as partial and complete COI coding sequences, which can vary considerably across metazoan phyla.

The majority of retrieved references were resolved to the Family (*n* = 722) or Genus (*n* = 431) taxonomic ranks, with fewer at the Order (*n* = 190), Class (*n* = 107) and Species (*n* = 53) levels, and with one reference identified at the Clade, and 14 at the Tribe level (Figure 2E; Tables S3 and S5).

### 3.3 Dual Pre‐Processing Sequence Data

**FIGURE 3:**
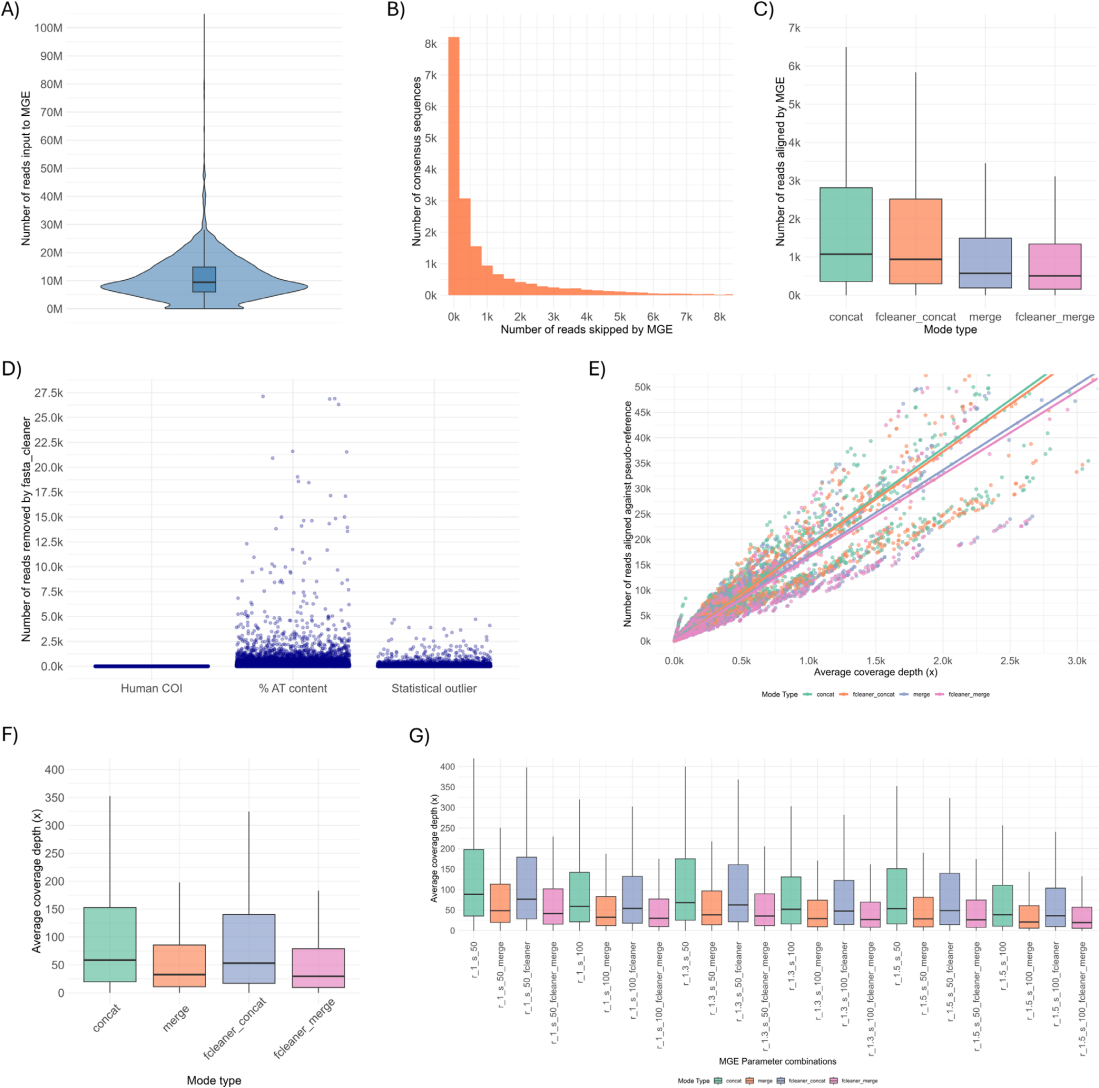
Metrics generated from 34,492 barcode consensus sequences produced from 1518 historical DNA genome skims, run through the BeeGees pipeline. (A) Number of million quality controlled/pre‐processed reads input into MitoGeneExtractor (MGE), (B) Number of reads skipped by MGE, (C) Number of reads aligned to protein pseudo‐references by MGE for each ‘mode type’, (D) Number of reads removed during each of the applied fasta_cleaner steps, with inlaid human COI‐filtered reads, (E) Scatter plot of average coverage depth (x) against number of reads aligned to protein pseudo‐references for each mode type, (F) Average coverage depth partitioned by ‘mode type’, and (G) Average coverage depth of barcode consensus sequences generated using each of the 24 MGE parameter combinations.

Pre‐processing via fastp removed an average of 432,732 reads due to insufficient length (< 15 bp), an average of 130,394 resulting from low quality (> 40% bases < Q15), and an average of 1614 reads for excessive ambiguous base calls (> 5 ambiguous bases). The retention rate following quality control varied considerably between processing modes due to the nature of read merging, where ‘concat’ mode resulted in an average of 85.31% (range: 13.78%–100%) and ‘merge’ mode samples 43.19% of reads retained (range: 3.13%–50%) (Figure 2F). Quality filtering substantially improved sequence quality metrics, with an increase in mean Q30 rate from 86% to 93%. The additional quality filtering and trimming undertaken for PE ‘concat’ mode reads resulted in an average loss of 6.51% reads (range: 0%–100%), amounting to an average of 14.46 M reads being retained for ‘concat’ mode parameter sets (Figure 2G). Contrastingly, ‘merge’ mode preserved an average of 7.5 M reads (range: 2–144.9 M), reflecting an approximate 50% reduction in reads input into MGE due to the merging process (Figure 3F; Table S5).

### 3.4 Barcode Recovery and Consensus Cleaning

Across the 1518 samples, MGE generated 18,216 barcode consensus sequences from 6 MGE ‘r’ and ‘s’ parameter combinations and 2 pre‐processing modes. Trimmed reads input to MGE ranged from 2 to 217.9 M (average: 11.0 M) (Figure 3A; Table S5). Of these, an average of 3074 reads aligned successfully to pseudo‐references (range: 0–412,439), with 1178 reads omitted by MGE due to alignment ambiguity, frameshifts, or low relative alignment (‘r’) scores (Figure 3B).

Following the sequential cleaning of MGE‐aligned reads via the fasta_cleaner module and the inclusion of 12 additional parameter combinations as a result, a further 16,276 ‘cleaned’ consensus sequences were generated. The cleaning process, which removed sequences with similarity to human COI, divergent AT content, or those that were statistical outliers, resulted in a total of 34,492 barcode consensus sequences across all 24 parameter combinations. Compared to pre‐cleaned barcode consensus sequences, those generated after cleaning showed an overall reduction in the number of reads aligned and used to construct the barcode consensus sequences by 9.51%, with an average of 2935 reads aligned per sample (Figure 3C). On average, ~0.01 reads per sample were removed due to human COI similarity, 233 reads (range: 0–40,330) due to divergent AT content, and 46.89 reads (range: 0–4271) were excluded as statistical outliers (Figure 3D).

Coverage depth was typically very high, averaging 179.29× for pre‐cleaned barcode consensus sequences, although it varied widely among samples (range: 0–19,832.02×) (Figure 3F,G). Cleaned consensus sequences comparatively displayed a slightly lower average coverage depth of 173.39×, a 5.9× reduction compared to pre‐cleaned barcode consensus sequences and equally had a considerable range across samples (range: 0–17,759.97×) (Figure 3F,G). A strong positive correlation was observed between the number of reads aligned and average coverage depth across all processing modes (*R* ^2^ = 0.916–0.959; Figure 3E). Overall, 91.6%–95.9% of the variance in coverage depth was explained by aligned read count, with ‘concat’ and ‘fcleaner’ modes demonstrating marginally stronger relationships (*R* ^2^ ~ 0.96) than merge‐based approaches (*R* ^2^ ~ 0.92), reflecting the decrease in aligned reads via ‘merge’ mode due to the merging process.

These results indicate that MGE's amino acid–guided alignment approach and associated barcode consensus sequence cleaning enabled effective barcode recovery across highly variable input read counts, aligned read numbers and read quality profiles.

**FIGURE 4:**
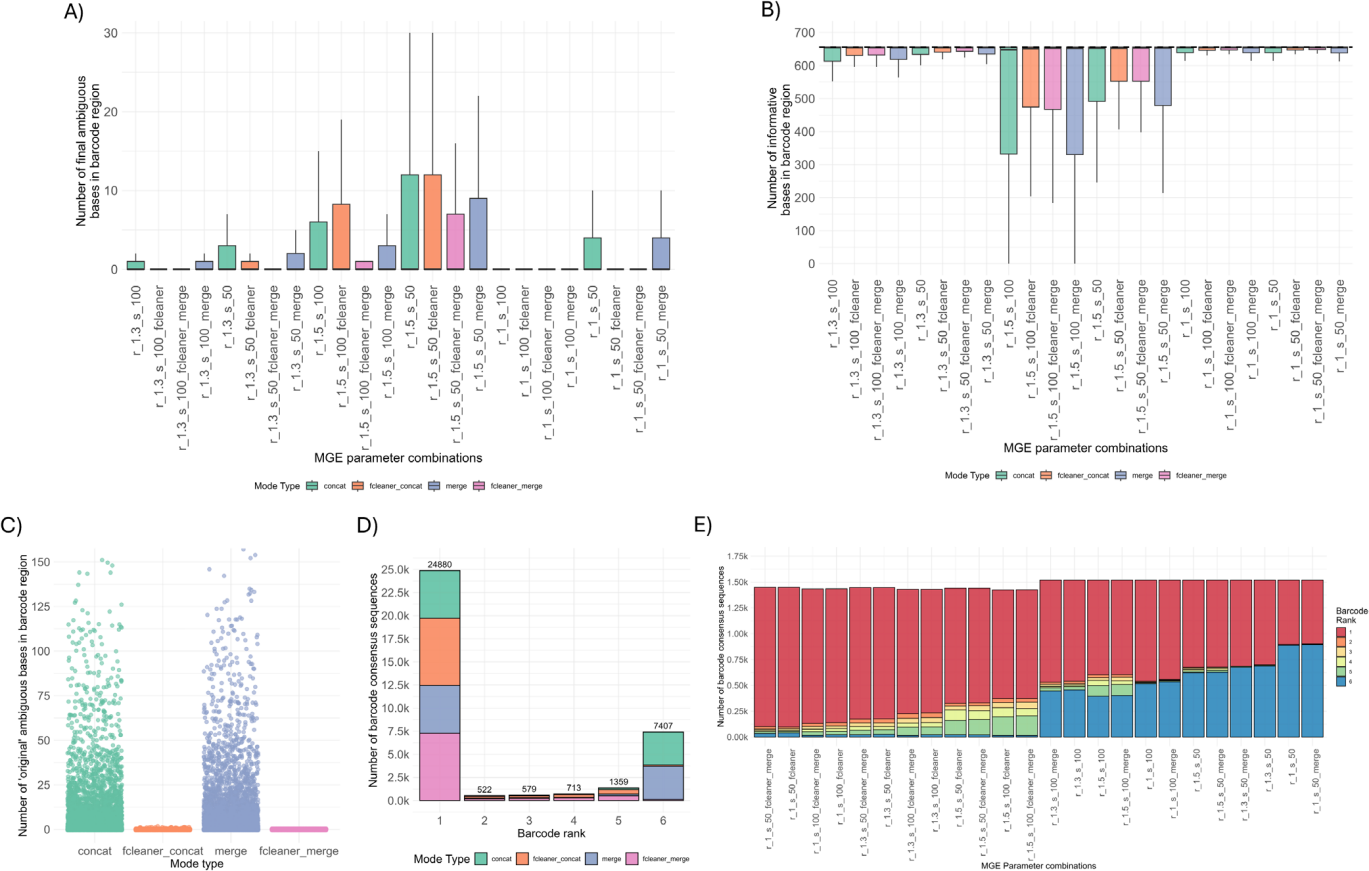
Structural validation metrics from 34,492 barcode consensus sequences produced from 1518 historical DNA genome skims, run through the BeeGees pipeline. (A) Number of ‘final’ barcode ambiguous bases across the 24 parameter combinations (i.e., number of ambiguous bases in barcode region prior to HMM processing (after tilde removal)), (B) Number of informative (non‐N) bases in barcode region across the 24 parameter combinations (dashed line at 656 bp maximum), (C) Number of final ambiguous bases in barcode region, grouped by ‘mode type’, (D) Number of barcode consensus sequences at each of the barcode rank (1–6) for each mode ‘type’ and (E) Distribution of barcode ranks (1–6) across the 24 parameter combinations.

The positive impact of the sequential cleaning process on barcode ‘quality’ was evident with nearly the complete elimination of ‘original’ N's (pre‐clean average: 4.98, post‐clean average: 0.0016) (Figure 4C; Table S5). Notably, pre‐cleaning sequence generated with the most permissive MGE parameters, ‘r_1_s_50_merge’ and ‘r_1_s_50’, exhibited the highest average number of ‘original’ N's (13.49 and 13.33 Ns, respectively), whilst the 12 ‘fcleaner’ parameter combinations contained the fewest ‘original’ N's of the 24 parameter combinations (0–0.005), underscoring the necessity of the cleaning step before final consensus generation when using relaxed parameters in MGE (Figure 4C).

Consensus sequence quality metrics varied systematically across parameter combinations. The average number of post‐HMM extraction, ‘final’ N base positions was 7.53 (range: 4.4–11.56), where a minimal reduction between pre‐ and post‐cleaned consensus sequences was observed (average of 7.87 and 7.17 ‘final’ N's, respectively). Similarly to ‘original’ N's, ‘r_1_s_100_fcleaner_merge’ sequences contained the fewest ‘final’ N's (4.4) and ‘r_1.5_s_50’ containing the most (11.56), reinforcing the positive impact of consensus cleaning and highlighting that consensus sequences constructed with lower stringency threshold tend to contain more N positions than more stringent parameter combinations (Figure 4A).

The average informative base count across all consensus sequences was 571.29 bases (range: 0–656), with ‘r_1_s_50’ for both ‘fcleaner’ and ‘fcleaner_merge’ yielding the highest values (623.1 and 623 bp, respectively), whilst ‘r_1.5_s_100_merge’ and ‘r_1.5_s_100’ produced the lowest (505 and 505.5 bp, respectively). This reflects the inclusion of greater numbers of reads at lower ‘r’ and ‘s’ value thresholds, but also the production of generally more informative barcodes post‐cleaning (Figure 4B).

### 3.5 Barcode Validation

Of the 34,492 barcode consensus sequences generated, 25,957 (75.3%) passed structural validation criteria and proceeded to taxonomic validation. The primary reasons for structural validation failure were due to insufficient barcode length (< 300 bp; *n* = 4238), the presence of N's in the original sequence (> 0 N's; *n* = 6041), excessive N content in the barcode region (> 30%; *n* = 2371), invalid reading frames (*n* = 1300) and the presence of internal stop codons (*n* = 107) (Table S5).

Barcode rank distribution was skewed towards rank 1 (72.1%; *n* = 24,880), followed by rank 6 (21.5%; *n* = 7407) and with ranks 2–5 collectively accounting for 6.4% (*n* = 3173) (Figure 4D). Parameter performance varied considerably, where ‘r_1_s_50_fcleaner_merge’ yielded the highest proportion of rank 1 barcodes, whilst ‘r_1_s_50_merge’ produced the lowest (Figure 4E). These results indicate that most consensus sequences met fundamental structural requirements for DNA barcodes, though parameter selection substantially influenced barcode quality and ranking.

BLASTn similarity searches of the 25,957 structurally validated barcodes against the BOLDistilled database yielded 2,563,215 hits (average: 98.7 hits per sequence). Following taxonomic matching (≥ 80% identity, ≥ 100 bp alignment length), 20,407 sequences (78.6%) successfully matched the input taxonomy. Of these, the majority matched at the species level (*n* = 474, 45.1%), followed by genus (*n* = 374, 35.6%) and family (*n* = 202, 19.2%).

**FIGURE 5:**
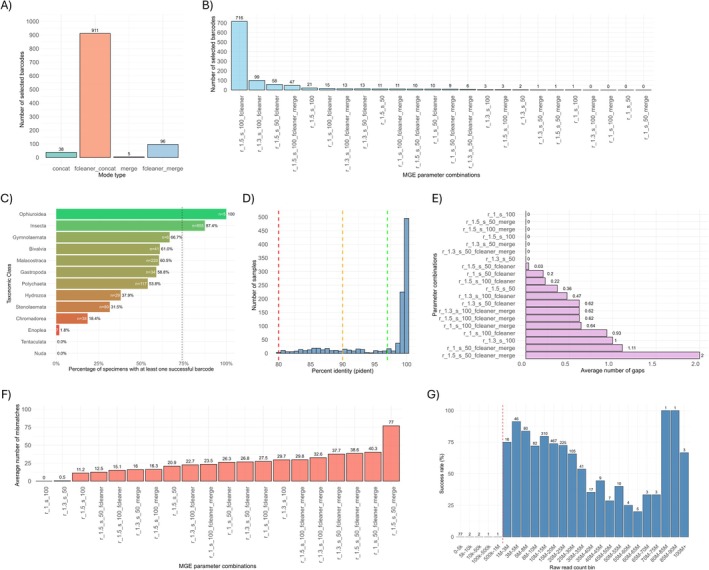
Taxonomic validation of barcode consensus sequences produced from 1518 historical DNA genome skims, run through the BeeGees pipeline. (A) Number of taxonomically validated, selected barcodes for each of the 4 ‘mode types’ (911 = 86.76%, 38 = 3.62%, 96 = 9.14%, 5 = 0.48%), (B) Number of selected barcodes across each of the 24 parameter combinations, (C) Barcoding success rate per taxonomic Class (only Classes with > 5 specimens (=13 classes, 1507 samples represented)), (D) Distribution of percent identity of the best hit for each selected barcode (*N* = 1050), (E) Average number of gaps throughout the best hit across each of the 24 parameter combinations (including zero's), (F) the average number of mismatches throughout the best hit across each of the 24 parameter combinations (including zero's) and (G) barcode validation ‘success’ rate per sample across 25 bins of raw PE read count, where the red dashed x‐intercept represents the ‘sequencing failure’ threshold.

Top hit sequence identity for each sample showed high‐quality matches, with 780 sequences (74.3%) exceeding 97% identity (Figure 5D; Table S5). Average alignment length was 563 bp, with average mismatch and gap counts of 18.41 and 0.48, respectively. Seven parameter combinations produced zero gaps across all barcode consensus sequences, whilst ‘r_1.5_s_50_fcleaner_merge’ had the highest gap count (average: 2) (Figure 5E). A single parameter combination, ‘r_1_s_100’, contained no mismatches across all barcode consensus sequences, whilst ‘r_1.5_s_50_merge’ contained the most mismatches with an average of 77 (Figure 5F).

Validated barcode selection varied substantially across parameter combinations (Figure 5A). Cleaned barcode sequences accounted for 1007 of the validated barcodes compared to only 43 pre‐cleaned sequences (Figure 5A). The most ‘successful’ combination was ‘r_1.5_s_100_fcleaner’ (*n* = 716), followed by ‘r_1.3_s_100_fcleaner’ (*n* = 99) and ‘r_1.5_s_50_fcleaner’ (*n* = 58), whilst four combinations (‘r_1_s_50_merge’, ‘r_1_s_50’, ‘r_1_s_100_merge’ and ‘r_1.3_s_100_merge’) yielded no validated barcodes (Figure 5B).

Overall, validated COI barcodes were generated for 1050 of 1518 samples (~69%) (Figure 5B; Table S5). Samples with < 1 M raw reads (deemed sequencing failures) systematically failed to generate validated barcodes (Figure 5G), although median raw PE read count did not differ significantly between successfully validated (~16.26 M) and those that failed validation (~17.07 M). When excluding these sequencing failures (*n* = 83), barcoding success rate increased to 73.17%. Barcoding success varied across taxonomic Classes (Figure 5C). Ophiuroidea showed 100% recovery (5/5), while Insecta, the most extensively sampled Class (*n* = 855), achieved 87.4% success. The remaining 11 Classes had success rates below the average. The comb jellies, Tentaculata and Nuda, were the only taxonomic Classes to have 0 successfully recovered and validated barcodes.

### 3.6 Predictors of Barcode Recovery Success

**FIGURE 6:**
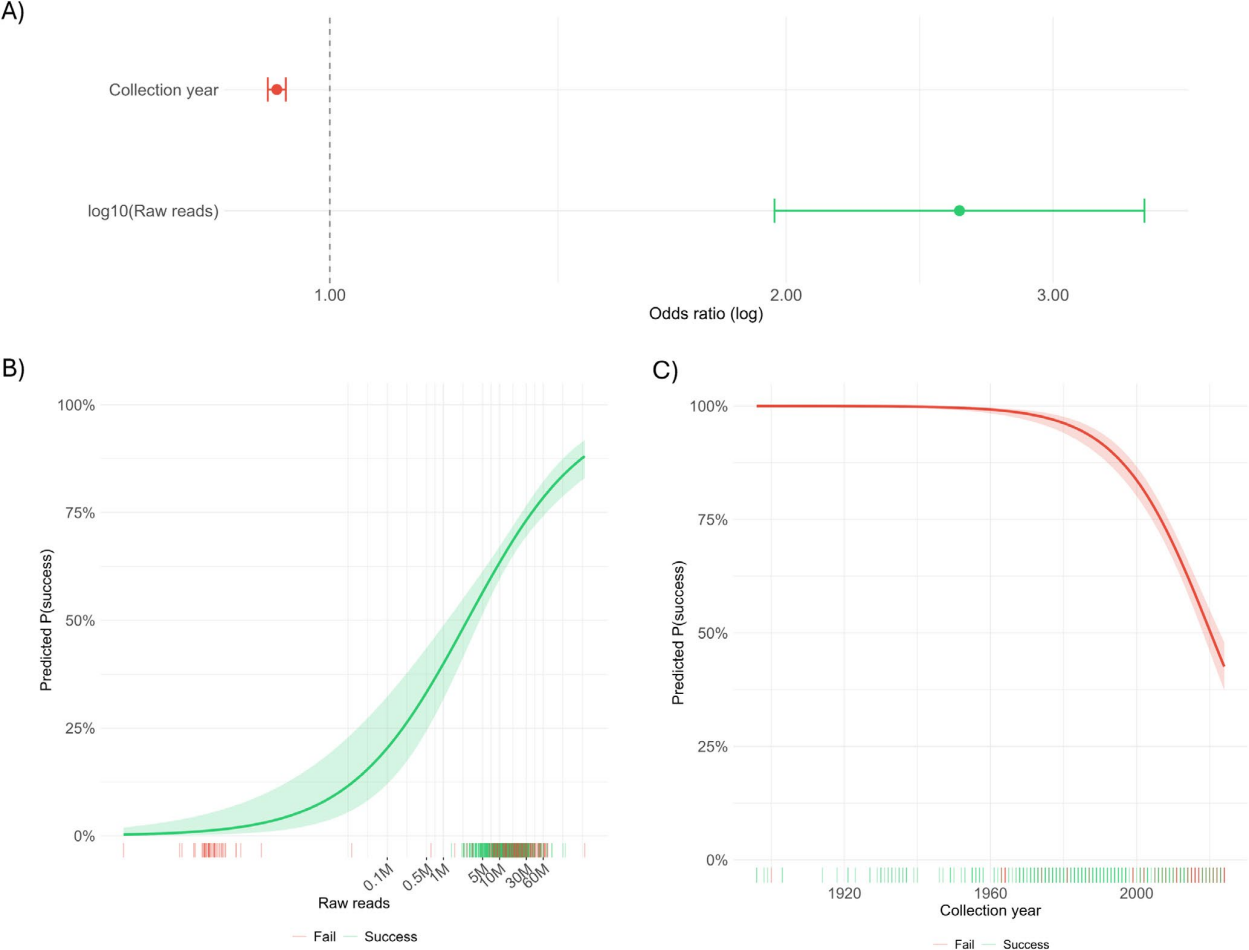
Logistic regression analysis of factors influencing COI barcode recovery success across 1037 genome skims. (A) Odds ratios (OR) with 95% Wald confidence intervals for collection year and raw read count predictors on a log scale. The dashed vertical line denotes OR = 1 (no effect). (B) Predicted probability of barcode recovery success as a function of raw read depth, with collection year held at the median (2011). The shaded ribbon represents the 95% confidence interval. Tick marks along the x‐axis indicate individual samples, coloured by outcome (green: Success; red: Failure). (C) Predicted probability of barcode recovery success as a function of specimen collection year, with raw read depth held at the median (16.1 M reads). Shading and tick marks are as in (B).

To explore the major factors influencing barcode recovery success, a binomial logistic regression model was fitted with log10‐transformed raw read count and specimen collection year as predictors. Both predictors were found to be highly significant, where a strong positive effect on recovery success was observed for raw read count (OR = 2.60, 95% CI: 1.97–3.45, *p* = 2.5 × 10^−11^) (Figure 6A), indicating that each 10‐fold increase in sequencing depth approximately tripled the odds of successful barcode recovery. Predicted success probability rose steeply between approximately 1 M and 20 M reads, with diminishing returns above this threshold (Figure 6B). Collection year showed a significant negative association (OR = 0.92 per year, 95% CI: 0.91–0.94, *p* < 2 × 10^−16^) (Figure 6A), such that more recently collected specimens were observed to have marginally lower predicted success rates when raw read count was held constant (Figure 6C).

## 4 Discussion

### 4.1 High‐Throughput Barcode Recovery From Historical Museum Specimens

The accelerating biodiversity crisis requires comprehensive molecular characterisation of natural history collections to embed future DNA‐based identification within the existing Linnaean framework and provide baseline population genetic measures to study the genomic impacts of species declines. The BeeGees pipeline helps unlock the molecular potential of these collections by enabling high‐throughput recovery of DNA barcode sequences from degraded museum specimens, providing both species identifications and supporting broader evolutionary and conservation genomic studies (Mullin et al. 2023). The pipeline was successfully applied to > 34,000 skimmed museum specimens within the BGE project. In the subset here analysed (1435 samples with > 1 M reads), BeeGees recovered and validated COI barcodes for 1050 specimens (73.2%), demonstrating robust performance across diverse taxonomic groups and variable data quality.

The computational efficiency of BeeGees represents an advancement for museomic workflows. Processing 1518 genome skims required approximately 120 h on HPC infrastructure, equivalent to under 5 min per sample, including quality control, barcode recovery, cleaning, validation and selection. The modular Snakemake architecture ensures reproducibility, automatic error handling and efficient resource allocation, which are critical for processing heterogeneous datasets where individual samples may fail at different pipeline stages.

### 4.2 Strategic Parameter Optimisation Maximises Barcode Recovery

A key innovation of BeeGees is the systematic exploration of multiple parameter combinations through automated per‐sample parameter sweeps, rather than reliance on a single fixed configuration. The pipeline evaluated six MGE parameter sets (*r* = 1.0, 1.3, 1.5; s = 50, 100) across two preprocessing modes (‘concat’, ‘merge’). With consensus cleaning, this led to the generation of up to 24 consensus sequences per sample prior to barcode selection through structural and taxonomic validation. Barcode recovery varied markedly across parameter space, demonstrating that optimal performance requires careful consideration of multiple interacting factors. As such, this brute‐force strategy accommodating for the highly variable quality of hDNA‐derived sequence data proved imperative. Twenty parameter combinations produced validated barcodes, with the most successful (r_1.5_s_100_fcleaner) yielding 716 validated barcodes, demonstrating that no single parameter set performs optimally across all specimens.

The relationship between parameters and sequence quality reveals important optimisation considerations. Lower ‘r’ values and ‘s’ values increased read inclusion and consensus length but also elevated sequence errors, consistent with chimeric alignments. Whilst s = 50 improves recovery from low‐coverage samples by relaxing alignment stringency, it increases susceptibility to contaminant read incorporation. Accordingly, non‐cleaned barcode sequences generated with ‘r_1_s_50’ and ‘r_1_s_50_merge’ exhibited the highest original ambiguous base counts, indicating chimeric consensus formation from mixed target and non‐target reads.

### 4.3 Read Count and Specimen Age as Predictors of Barcoding Success

Conventional sequencing typically assumes that greater read depth improves assembly quality and genome completeness. However, barcode recovery from the 1518 museum genome skims analysed here shows a more complex relationship. Samples with fewer than one million reads consistently failed to yield validated barcodes, defining a practical sequencing threshold, yet median read counts did not differ significantly between successful and unsuccessful samples. Furthermore, barcode recovery success exhibited a non‐linear pattern, with reduced success at intermediate depths (35–75 million reads) and higher recovery at both lower (1–35 million) and higher (> 75 million) read counts, although sample numbers were low at intermediate read counts. This apparent paradox reflects the multifactorial determinants of museomic barcoding success, including genome size, endogenous DNA content, preservation state, read length distribution and reference availability. High read counts dominated by contaminant DNA will yield fewer informative reads than lower read count samples with higher endogenous content, while extremely short, degraded fragments (≤ 30 bp) may align poorly despite abundant sequencing.

Logistic regression confirmed these observations, supporting raw read depth as a significant independent predictor of success (OR = 2.60 per 10‐fold increase). The model additionally identified a negative association between collection year and recovery success (OR = 0.92 per year), which is likely confounded by taxonomic composition and preservation state rather than reflecting a direct, causal effect of specimen age on DNA quality. For example, many of the more recently collected specimens in the dataset are marine invertebrates, which notoriously have large and complex genomes.

The absence of a universal read‐count threshold for success further emphasises the value of BeeGees' parameter‐sweeping approach, which adapts to each sample's specific quality profile rather than applying uniform processing across taxonomically and preservation‐diverse collections.

### 4.4 Consensus Cleaning Improves Barcode Quality

The marked improvement in sequence quality following the application of fasta_cleaner highlights the metagenomic nature of genome skims from museum specimens. Historical DNA routinely comprises mixtures of degraded target DNA and multiple contaminant sources, including modern handling contaminants, co‐preserved organisms (e.g., gut contents, parasites, symbionts) and environmental microbiota. Without filtering, these reads can generate aforementioned chimeric consensus sequences through misalignment to the reference.

The sequential filtering approach implemented in fasta_cleaner systematically removes these contaminants, eliminating all original ambiguous bases and generally lowering final ambiguity, demonstrating that the removed sequences represented genuine contaminants rather than target variation.

An optional BWA‐based reference filtering extends this capability by enabling user‐defined removal of reads, facilitating targeted control of known cross‐contamination from complex genomic backgrounds. This flexibility proves particularly valuable when processing samples with expected contaminants (e.g., parasitoid insects preserved within hosts, gut contents, or specimens stored in close proximity).

### 4.5 Reference Sequence Availability and Gap‐Filling Initatives

Gene Fetch retrieved COI protein references exceeding 500 amino acids for all 1518 samples, with most resolving to family (*n* = 722) or genus‐level (*n* = 431). The scarcity of species‐level references on GenBank reinforces the substantial gaps in database coverage across many taxa. Although more distant references are generally sufficient for protein‐guided alignment, they increase positional uncertainty and reduce sensitivity, particularly in lineages with high substitution rates or atypical codon usage. Furthermore, sparse reference coverage also limits taxonomic validation success due to fewer high‐confidence BLAST assignments.

These results further support the need for coordinated reference gap‐filling initiatives, such as BGE. Accordingly, all validated barcodes generated here are deposited in BOLD (Table S4) to improve reference availability. Strategic sampling of poorly represented lineages will progressively reduce reference remoteness and enhance barcode recovery from museum collections.

### 4.6 Taxonomic Validation and the BOLD Taxonomy Framework

Structural validation removed 24.7% of barcodes prior to taxonomic validation. The stringent criteria applied, based on BOLD's (BIN) compliance standards, ensure that validated sequences meet international requirements for taxonomic identification and molecular systematics (Hebert et al. 2013).

Of the 25,957 structurally valid sequences, 20,407 (78.6%) were subsequently successfully taxonomically validated against the 1.7 million‐record BOLDistilled COI‐5P database, demonstrating robust performance of the hierarchical matching approach. The taxonomic assignment quality of the validated barcodes was generally high, with 74.3% of barcodes exceeding 97% sequence identity, 45.1% of which resolved to species‐level and alignments were typically complete or near‐complete with minimal gaps and mismatches. This reflects the broad representation of different lineages in the BOLDistilled database and thus supports its application to such approaches.

This performance reflects the value of curated, marker‐specific reference databases with robust taxonomic backbones. Here, the use of the BOLD taxonomy and BIN framework proved advantageous over NCBI‐derived resources by resolving synonymy and taxonomic ambiguity and by providing barcode‐specific, quality‐filtered references that minimise spurious matches. Equivalent benefits would be expected from similarly curated reference sets for alternative markers (e.g., rbcL), supporting the flexibility of the BeeGees pipeline across taxonomic groups.

### 4.7 Implications for Museum Collection Sequencing Initiatives

The overall barcode recovery rate (73%) provides a realistic benchmark for large‐scale museomic initiatives. Not all specimens will yield barcodes, regardless of sequencing effort, due to intrinsic preservation limitations leading to DNA degradation (suboptimal preservation media, fluctuating storage conditions, delayed fixation, or historical chemical treatments) and specimen age.

Recovery rates varied considerably across taxonomic groups, where *Insecta* achieved 87.4% success compared to substantially lower rates in marine invertebrate classes, reflecting the influence of taxon‐specific preservation, genome characteristics and reference database representation on overall pipeline performance. The identification of approximately one million reads as a practical minimum sequencing threshold provides actionable guidance for future initiatives, where below this depth, barcode recovery is unlikely regardless of pipeline choice or parameter optimisation.

This performance is nevertheless comparable to that of PCR‐based approaches applied to historical material, where Sanger sequencing success typically ranges from 20% to 75% depending on age and tissue type (Hebert et al. 2013; Hernández‐Triana et al. 2014; Mitchell 2015; Jaksch et al. 2016; Silva et al. 2019; Salis et al. 2025). Furthermore, PCR workflows frequently require multiple primer sets, greater DNA input and taxon‐specific optimisation, limiting scalability. Genome skimming, despite current higher per‐sample sequencing costs, enables parallel processing of thousands of taxonomically diverse specimens, recovery of complete or near‐complete organellar genomes (White et al. 2025) alongside specific barcode loci of interest, provides data suitable for phylogenomic and population genetic analyses beyond initial species identification and supports k‐mer–based analyses such as genome size estimation and other reference‐free genomic inferences. As sequencing costs further decline and reference databases expand, genome skimming is likely to become the standard approach for molecular characterisation of natural history collections.

### 4.8 Future Developments and Enhancements

Future development of BeeGees could include enhancements addressing deficiencies identified in this large‐scale benchmark. Contamination detection may be expanded through k‐mer based taxonomic profiling of filtered reads, generating summary reports of contaminant identity and abundance, enabling automated pre‐barcode recovery filtering. Such reporting could assist users in identifying systematic laboratory contamination and implementing corrective measures. Additionally, HTML summaries could improve reporting by consolidating key metrics and data visualisation, including success rates, parameter performance comparisons, coverage distribution statistics and sample‐specific outcomes (e.g., complete success, partial success with structural validation only, complete failure), thereby streamlining assessment across workflow runs.

Finally, the pipeline will add support for alternative protein‐coding genes, extending BeeGees' applicability, particularly across plant and fungal collections through integration of locus‐specific HMMs and validating them against established barcoding standards. In principle, this framework could also be extended to recover multiple mitochondrial PCGs simultaneously from a single genome skim, provided that appropriate protein references, locus‐specific HMMs and taxonomic validation databases are available for each target gene. The development of additional, high‐quality, curated reference libraries for use as BLAST databases is outlined in the BOLDistilled roadmap (Prosser et al. 2025) and will expand BeeGees' applicability beyond COI‐5P and rbcL. Whilst not reported here, BeeGees showed similar success when applied to rbcL extraction within the BGE project.

In conclusion, the BeeGees pipeline demonstrates that high‐throughput, quality‐controlled barcode recovery from degraded museum specimens is achievable at scale using genome skimming. Through systematic parameter exploration, rigorous contamination filtering and customisable validation criteria, the workflow establishes a practical benchmark for museomics. The pipeline's flexibility, reproducibility and computational efficiency position it as a valuable tool for biodiversity genomics and gap‐filling initiatives seeking to unlock the molecular potential of natural history collections.

## Author Contributions

D.A.J.P., R.A.V. and B.W.P. were involved in conceptualisation and review of initial and final manuscripts. D.A.J.P. wrote the manuscript draft and conducted all analyses. D.A.J.P., with input from R.A.V. and B.W.P., developed the BeeGees pipeline.

## Funding

The authors received funding from Biodiversity Genomics Europe (Grant No. 101059492), supported by Horizon Europe (Biodiversity, Circular Economy and Environment call), with co‐funding from SERI (22.00173 and 24.00054) and UKRI.

## Disclosure

Benefits Generated: The sequence data utilised here was generated through the Biodiversity Genomics Europe (BGE) project, derived from museum specimens contributed by partner institutions across Europe. All sequence data and associated results have been shared with the BGE consortium and are openly available to the broader scientific community (see Data Accessibility statement). The barcode data generated will directly support biodiversity monitoring, species identification and conservation efforts across Europe. This work reflects an ongoing commitment to open science and collaborative international research partnerships.

## Conflicts of Interest

The authors declare no conflicts of interest.

## Supporting information


**Table S1:** Higher taxonomic information (Phylum, Class, Order, Family) of 1518 museum specimens processed by BeeGees.
**Table S2:** Family‐level ranks within each of the 18 museum specimen Classes.
**Table S3:** Protein pseudo‐reference metadata for the 1518 processed specimens, produced by Gene Fetch integrated within BeeGees.
**Table S4:** Barcode Of Life Dataabase (BOLD) sample metadata, including Process and Sample Identifiers, BOLD Barcode inde Number (BIN) assignment, COI barcode length and ambiguous base content and BOLD taxonomic identificaiton information.
**Table S5:** Final BeeGees summary metrics output, including pre‐processing, Gene Fetch protein pseudo‐reference retrieval, MitoGeneExtractor, sequential barcode consensus cleaning and structural and taxonomic validation metrics for the 36,432 barcode consensus sequence generated from the 1518 museum specimens used here to benchmark BeeGees. Please refer to the BeeGees GitHub repository for more detailed information on each metric within this spreadsheet.

## Data Availability

The pipeline used to generate the findings of this study are openly available at https://github.com/bge‐barcoding/BeeGees, and also archived at https://zenodo.org/records/18803222, reference number https://doi.org/10.5281/zenodo.18803221.
